# Role of miRNAs interference on ovarian functions and premature ovarian failure

**DOI:** 10.1186/s12964-022-00992-3

**Published:** 2022-12-23

**Authors:** Narjes Nouri, Olduz Shareghi-Oskoue, Leili Aghebati-Maleki, Shahla Danaii, Javad Ahmadian Heris, Mohammad Sadegh Soltani-Zangbar, Amin Kamrani, Mehdi Yousefi

**Affiliations:** 1 Student Research Committee, Tabriz University of Medical Sciences, Tabriz, Iran; 2 Stem Cell Research Center, Tabriz University of Medical Sciences, Tabriz, Iran; 3 Department of Immunology, School of Medicine, Tabriz University of Medical Sciences, PO Box: 516-6615573, Tabriz, Iran; 4 Immunology Research Center, Tabriz University of Medical Sciences, PO Box: 516-6615573, Tabriz, Iran; 5 Gynecology Department, Eastern Azerbaijan ACECR ART Center, Eastern Azerbaijan Branch of ACECR, Tabriz, Iran; 6 Department of Allergy and Clinical Immunology, Pediatric Hospital, Tabriz University of Medical Sciences, Tabriz, Iran; 7 Research Center for Integrative Medicine in Aging, Aging Research Institute, Tabriz University of Medical Sciences, Tabriz, Iran

**Keywords:** Premature ovarian failure, Effective micro-RNA in ovary, Effective signaling in the ovary

## Abstract

Premature ovarian failure is a to some extent unknown and intricate problem with diverse causes and clinical manifestations. The lack of ovarian sex hormones presumably is effective in the occurrence of ovarian failure. Our progress in this field has been very little despite undertaken scientific research endeavors; scholars still are trying to understand the explanation of this dilemmatic medical condition. In contrast, the practice of clinical medicine has made meaningful strides in providing assurance to the women with premature ovarian insufficiency that their quality of life as well as long-term health can be optimized through timely intervention. Very recently Scientists have investigated the regulating effects of small RNA molecules on steroidogenesis apoptosis, ovulation, gonadal, and corpus luteum development of ovaries. In this literature review, we tried to talk over the mechanisms of miRNAs in regulating gene expression after transcription in the ovary.

**Video abstract**

**Supplementary Information:**

The online version contains supplementary material available at 10.1186/s12964-022-00992-3.

## Introduction

MicroRNAs (miRNAs) are a class of small non-systematize RNAs that function in gene regulation accompanying a main role in cell activity, proliferation, and development [1]. The supervisory part of these small RNA molecules has currently surveyed in ovarian cells, uncovering their influence on steroidogenesis, gonadal development, ovulation, apoptosis, and corpus luteum development. Herein, we reviewed the current understanding of miRNA biogenesis, mechanism, and the act that miRNAs play in gene expression regulation following transcription, as well the current evidence of miRNAs practice in ovarian development and function [2]. Ovulation and oocyte growth happens inside the ovaries. Ovaries granulosa cells provide estrogen for ovaries itself and also endocrine signals to other organs and tissues [3]. Ovarian somatic cells work for the growth and maturation of the oocyte very near to the rise of luteinizing hormone (LH), creating a physiological reaction above ovulation. This answer involves, encouraging steroidogenesis, meiosis, follicular maturation, cumulus cells development, progesterone secretion, and luteinizing, eventually oocyte maturation [4, 5]. It has been discovered that Dicer (ribonuclease III) knockout, which is in charge of pre-small RNA processing into functional small RNAs caused disruption in the several biological events inside the ovary including folliculogenesis, oocyte development, ovulation leading to infertility [6–10]. siRNAs, miRNAs, and piRNAs are the principal small RNAs work for the healthy functioning of the ovary. PiRNAs mainly pay to the heathy functioning of the germ cell [11]. With regard to complicated miRNAs play during fetal development, fetal gonadal steroidogenesis includes many genes linked expression [12].

Premature ovarian failure is a intricate disease with prevalence of 1 in 250 women below the age 35 and 1 in 100 women below 40 [13–15]. POF characteristics consist of increased estrogen level, decreased gonadotropin hormone level, lack of menstruation that pay to female infertility and medical condition prior to the menopause [16]. Additionally there is some adverse outcomes to POF including heightened probability of cardiovascular diseases, weakened sexual function and fragile bones [16]. Three categories of cells are found in Ovarian follicles: oocytes, granulosa and theca cells. Follicle-stimulating hormone (FSH) and luteinizing hormone (LH) affect granulosa and theca cells through specific receptors, which are vital for the follicles growth and development and this normal folliculogenesis process is changed in POF [17, 18].

Scientific investigations have identified the role of gene defects like X chromosome and autosomes abnormalities in the POF emergence, particularly structural differences and X chromosome translocation with autosomes trisomy of X, Turner syndrome, pre-mutations and mutations of X associated genes and also aberrations of autosomal associated genes have been detected in POF cases [18, 19].

In addition to genetic and chromosomal parameters interfering in the appearance of POF, other determinants are including autoimmunity, enzymes, and environmental factors [18, 20].

## Profile of effective miRNAs in the ovary

miRNAs are expressed in the ovary function for the managing of mammalian reproduction. miRNA expression profiles have recognized in various species containing human, mouse, bovine, sheep, chicken, fish, swine and equine species [21]. miRNA expression in each organ is closely related to organ function. The expression and function of miRNAs depend on the cell types is different. The ovary contains oocytes and many somatic cell types such as granulosa cells, theca cells, and cumulus cells. In total, 58 miRNAs were mainly expressed in bovine fetal ovary compared to somatic tissue. Eight miRNAs bta-miR-10b, bta-miR-99a, bta-miR-199a-5p, bta-miR-199a-3p, bta-miR-100, bta-miR-424, bta-miR-214and bta-miR-455) expression were 10 times higher in fetal bovine ovaries than in the pool of somatic tissues. Additional examination indicated that bta-miR-10b together with bta-miR-424 were extremely amplified in oocytes germinal vesicle (GV)[22]. Analogous expression arrays proposed to the maternal inheritance of these miRNAs and might possibly stay engaged in transcription throughput zygote gene activation. Numerous miRNAs function in maturation of oocyte, like miR-7, miR-2, miR-100, miR-184, miR-9b, let-7, miR-133, miR-79, miR-252 and miR-275 indicating different miRNA expression in several stages of the egg [23–25]. miR-133 and miR-2 are meaningfully amplified in metaphase I (MI) compared to GV stage, and both prevent cyclin B translation through abridging the 3′-UTRs of the crab cyclin B gene [24]. Several miRNAs, like the let-7 family, showed a species-independent housekeeping character in the ovary [26, 27]. Granulosa cells express miRNA differentially during luteal and follicular stages. miR-503 expression decreased in both the luteinization and FSH-reactive follicular growth stages however increased in the following pre-ovulatory stage [6].

Xu et al. characterized the cumulus granulosa cells of human (CGCs miRNA profile), and introduced let-7 family as the profusely amplified miRNA in cumulus granulosa cells of both women with healthy cycling and women with polycystic ovarian syndrome (PCOS) [27]. Comparing to the women with heathy cycling, the expression of miR-1307-3p, miR-10a-5p, miR-1273g-3p, miR-423-5p, miR-185-5p, miR-199a-3p, and miR-483-5p intensely boosted in the GC cells of women with PCOS. miR-483-5p expression overpowers both mitogen-activated protein kinase 3 (MAPK3) and Notch3 with decreasing expression in CGCs through direct bounding to MAPK3 and Notch3 mRNAs 3′-UTRs [27]. Bioinformatics and Gene regulation studies have discovered the effective sequences of pronouncedly expressed miRNAs in the ovary which contributing in the cellular events including cell proliferation, cell cycle, and apoptosis affecting ovaries functioning; and endocrine arrangement [28]. Moreover, recently it has been revealed that miR-143 prevents early follicle development through decreasing cyclin-dependent kinases (CDKs) 4 and 6 as well B1, D2,and E2 cyclins expression in early granulosa cells [29]. Additionally, miR-181a prevents the mice ovaries granulosa cells growth through direct impacting of activin IIA receptor [30]. miR-26b impedes the ataxia telangiectasia gene mutation (ATM) and in follicular ovaries granulosa cells promotes apoptosis [31]. In conclusion, miR-212 and miR-132 do some touches on ovulation and luteinization through endocrine system regulation [32].

## miRNAs and ovarian function

### miRNAs impact on ovarian follicle development

Depending on the species, the oocyte can remain suspended in the primordial follicle in the prophase of meiosis 1 for months and years. Follicle growth is initiated by cyclic signals with unknown origin. At this stage, the egg creates a thick extracellular matrix that forms the zona pellucida, which leads to the relative separation of the egg from the pre-granulosa cell layer. At this stage, pre-granulosa cells are differentiated into cubic granulosa cells and begin to multiply. Follicle growth continues with successive growth of oocyte and granulosa cells. The granulosa cells fluid-filled antrum is created among the granulosa cells layers (antral follicle). At the same time that granulosa cell and oocyte growth carry on, the granulosa cells excrete follicular liquid, leading to the creation of the antral hollow in the widening follicle. This vital liquid contains of proteins and other molecules providing nutrition as well signaling molecules among dissimilar cells inside of the follicle. The antrum formation encouraged granulosa cells departure and differentiation into cumulus cells. The cumulus granulosa cell layer is the oocyte nearby layer, maintaining straight interaction with oocyte by means of trans-zonal reactions spreading from cumulus cells in order to contact the oocyte surface in the porous zona pellucida. Cumulus cells and the oocytes impact each other through autocrine elements, gap junctions, and probably with transference of tiny extracellular vesicles (exosomes/micro-vesicles), sharing molecules among the granulosa cells and the oocytes seems indispensable for the follicle’s maturation, since either oocyte or the granulosa cells could not last by their own. Since these cells are dependent to each other, the gene expression regulation in both cells is vital for emerging the healthy babies. Numerous research work has confirmed the differential expression of miRNAs in diverse sections and time points inside the ovary, including oocyte development, luteal action and follicle maturation [33].

During different phases of follicle development, diverse growth elements indicate dissimilar properties on phase-specific purposes in disparate cell categories [2, 34]. As well miRNAs critically function in the different phases of follicle development, covering tiny follicles (1.5–3.5 mm), average follicles (4.0–5.5 mm), pre-ovulatory follicles, early corpora lutea, late corpora lutea, and corpus albicans. The utmost profusely expressed miRNAs through the different stages of development are miR-125b, miR-21, let-7a, let-7b and follicular stage over-expressed miRNAs are miR-145, miR-199a-3p and miR-31 with noticeable reduction in the follicular-luteal transformation. In the contrary, miR-21, miR-142-3p, and miR-503 are expressed at minor quantities in the follicular periods with marked rises in luteinized tissues [35]. Based on a study, miR-181a expression decreased in mice pre-antral and antral follicles relative to mature follicles similarly miR-181a inhibited activin receptor IIA expression (acvr2a) as well reduced the phosphorylation of intracellular signal transducer activin and Suppressor of Mothers against decapentaplegic homologue 2 (Smad2) in rat granulosa cells, affecting the granulosa cell proliferation and ovaries development [30]. Folliculogenesis starts with collapsing clusters of germ cells and primary follicles formation. Zhang et al. with in situ hybridization presented that pre-granulosa cells express miR-143 and miR-143 which hinders primordial follicles formation through pre-granulosa cell proliferation suppression and declining cell cycle-specific genes expression, like cyclin D2, CDK6, and CDK4 [29]. Since, across folliculogenesis, above 99% of ovarian follicles turn into atresia, miRNAs function in the follicle growth and atresia have lately been recognized. Since, disparate miRNAs expression is designed for healthy, pre-atretic and more atretic follicles [31] For this reason, P-miR-1281, Hsa-miR-936, mmu-miR-1224, hsa-miR-26b, P-miR-466g-b, hsa-miR-10b, P-miR-1275, R-miR-26b, hsa-miR-574-5p, hsa-miR-1275, hsa-miR-149*, and hsa-miR-99a a miRNAs expression increased, while hsa-let-7i, R-let-7a, hsa-miR-92a, hsa-miR-92b, P-miR-923, R-miR-739, hsa-miR-1979, hsa-miR-1826, hsa-miR-1308, P-miR-1826, and ssc-miR-184 expression decreased in follicles. Increased miR-26b expression, in follicular atresia, promotes DNA breaks and increases apoptosis in granulosa cells by direct objecting ATM and follicles trigger atresia through granulosa cells apoptosis [36, 37]. Considering miRNAs function in the trigger of apoptosis in granulosa cell, miR-34s initiates cell apoptosis and stops growth by p53 activation as well p21 cyclin-dependent kinase inhibitor [38, 39].

Tu et al. stated in a study on pig ovarian follicles that miR-34a encouraged apoptosis in granulosa cells through targeting beta B (INHBB) gene inhibition [40]. Carletti et al. stated that luteinizing hormone (LH) caused higher expression of miR-21 in rat granulosa cells and miR-21 silencing in vitro triggered apoptosis in granulosa cells [41]. Based on very advanced research findings miRNAs play key role in the oocyte maturation and the eggs move in to the meiosis stage at the initiation of DNA synthesis and stays in the MI stage till meiosis continues. Prior to ovulation, oocytes turn in to secondary oocytes following the first meiosis and are stopped in meiosis metaphase II (MII) up to fertilization [7, 25]. Xiao et al. stated that transgelin 2 (TAGLN2), with encoding an actin protein, contributes to ovaries growth and maturation. Furthermore, miR-133b controls oocyte development with targeting TAGLN2 at both mRNA and protein producing levels [25]. Dicer as a ribonuclease works in the synthesis and production of functional mature miRNAs in both granulosa cells and oocytes of the mice ovaries follicles [35].

Similarly, Dicer work in pre-ovulation follicles development has been clarified. Lei et al. stated that temporary inactivating of Dicer1 in follicular granulosa cells caused augmented supply in the pool of primordial follicles, hastened early recruitment of follicles and an upsurge in corrupt follicles in ovaries with temporary Dicer knockout (cKO) [6].

miR-503 is an ovary-specific miRNA, Dicer1 affects follicle maturation by knockdown of miRNA and miR-503 sequences. Inactivating Dicer1 in female rats led to atypical follicles formation and infertility [6, 7, 42].

Based on research studies Dicer functions importantly in follicle growth and oocyte maturation. Number of elements like members of TGF-β superfamily [43, 44], Ligand stimulating of type I activin receptor-like kinases (ALKs) and Smads [45–47] are regulated by miRNAs impacting follicle development. Recently in a study has been revealed that miR-224 expression regulates TGF-β/Smad signaling, miR-224 overexpression promotes TGF-β1-mediated granulosa cell proliferation together with Smad4, whereas miR-224 suppression partly overpowers TGF-β1-mediated granulosa cell proliferation, representing critical work of miR-224 in folliculogenesis [48].

### Secondary and early antral follicles miRNA content

Because of the need for isolation of small follicles out of pure populations and inadequate available procedures for ex vivo studying of folliculogenesis, a few investigations have been advocated for studying miRNAs role in tiny and developing follicles, moreover, even though there are range of molecular protocols for optional silencing of miRNA genes in early follicles development, most of the trials are intricate and often subject to fail also due to the miRNA progressive regulatory abilities, certain knockout could not abolish the preceding synthesized miRISC, thus fail to change function. Consequently, large part of ongoing research inspecting miRNA properties in folliculogenesis could be introductory in vitro inferences, clarifying some of the inconsistent data driven from the studies presenting proof of miRNA in vitro expression contribution in variable facets of granulosa cell function [34, 49–51]. Majority of the ovarian investigations largely depend on the cultivated granulosa cells or immortal granulosa cell lines, even though lack of in vivo functional studies always has been felt. Findings of in vitro experimentations without specific linking to studied in vivo effects cannot either specify the endogenous in vivo position, or justify the miRNAs properties and require precise validation and confirmation in order to be adequately comprehended. Early scientists’ inferences could provide to some extent the outline for upcoming research and medical practice either with confirming or discrediting the impact of these small RNA molecules in ovarian biology. Since, it is of a highly importance to verify many facets of inter- and intracellular complex signaling, in this way, in vitro experiments can be major implements to aid deciphering the codes of cellular communications. One example can be the constant study of transforming growth factor (TGF) regulation mediated miRNAs signaling pathway. TGF-β1 superfamily Proteins function critically in follicles’ maturation, and related miRNAs regulate this complex signaling pathway. Yao et al. revealed that miR-224 expression affected 16 TGF-β1 reactive miRNAs detected in cultured granulosa cells of pre-antral murine [48]. Based on bioinformatics studies, smad4, a TGF-β1 signaling intracellular effector [52], previously recognized as a miR-224 potential target. Increased expression of miR-224 declined the protein amounts of smad4 in cultivated granulosa cells, though it indicated a slight influence on mRNAs expression [48, 53]. Regarding GABRE as a TGF-β1 reactive gene, p53 and p65 are tumor suppressor genes regulate GABRE and miR-224 and p53 and/or p65 Knockdown leads to boosted amounts of miR-224 as well higher granulosa cell proliferation through TGF-β1 signaling [54]. A current study verified that miR-224 connects to the pentraxin 3 (ptx3) from 3'UTR head, a gene which is vital for cumulus cells growth [55]. It is assumed that after LH stimulation miR-224 expression should be reduced however the findings of these studies displayed irrelevant alterations in miR-224 level. This research claimed that TGF-β1 instigated a cut in miR-224 level, accordingly the amplitude in ptx3 and LH/hCG treated cumulus cells growth is inevitable [55]. Strangely, this finding opposes earlier findings indicating miR-224 rise responding to TGF-β1 in immature mice pre-antral granulosa cells [48].

### Antral follicles miRNA content

During development of primary antral follicles, luteinizing hormone receptor (LHCGR) level in granulosa cells is elevated through estradiol and follicle-stimulating hormone. The LH rise results in ovulation; therefore, this stimulation is indispensable for opting of the main follicle(s) and for ensuing LH-reactive molecular events within granulosa cells. The LH upsurge stimulates key modifications in genes function in pre-ovulation granulosa cells, resulted in modifications in several pathways inside the cells, comprising miRNAs, transcription factors, and matrix renovation factors [56, 57]. Alterations in miRNA expression after LH upsurge redirect general variations in gene expression [32].

Using a bioinformatics approach, Troppmann et al. analyzed the 3′UTR sequences of the LHCGR gene in search of miRNAs that might regulate its expression [58–60] Their analysis identified miR-513a-3p as a potential regulator of LHCGR. This miRNA was detectable in whole ovarian lysates in addition to human granulosa cells collected from large antral follicles of women undergoing assisted reproduction. Further test of the gene sequence for miR-513a-3p identified it as an X-linked gene appeared only in animals. To decide either there is a connection between the expression levels of miR-513a-3p and LHCGR, scientists measured the levels of both gene collected yields of human luteinized granulosa cells during oocyte retrieval in assisted reproductive technologies (ART) all along cultivation earlier calculated in lab environments [60].

During sample gathering, dropped LHCGR and heightened miR-513a-3p expression was observed. By time pass, levels of miR-513a-3p increased as LHCGR levels decreased. Thus, they demonstrated an inverse association between expression of LHCGR and miR-513a-3p, supporting the role of miR-513a-3p in affecting expression of LH receptor in granulosa cells [60].

miR-212 and miR-132 placed in 11700016P03Rik gene intron number 1 and thus both are contributed in the transcript of pri-mRNA. LH signaling causes cAMP-regulatory element-binding protein (creb) activation and Creb in neurons regulate miR-212 and miR-132 expression, therefore this could enlighten the reason of rise in miR-212 and miR-132 expression after LH activation [32, 61]. Bioinformatics survey has been performed with the aim of finding miR-132/212 miRNAs from the 3′UTR of ovarian mRNA databank and 77 sequences detected and recognized indicating ctbp1 as of a particular attention. Ctbp1 (carboxy-terminal binding protein 1) act together with steroidogenic factor 1 (sf-1), thus modulate promoter activity and regulate steroidogenesis in adrenal cells [62]. A comparable character however has not been confirmed yet suggesting for miR-132/212 in affecting steroidogenesis in granulosa cell. Granulosa cells stimulation by LH/hCG indicated no change in ctbp1 mRNA amounts, though meaningfully reduced ctbp1 protein amounts suggesting an activity for miRNA following transcription [32]. Recent data in bovine oocytes proposes that miR-212 regulates the oocyte-specific FIGLA expression [63]. Therefore, further studies are needed for miR-132/212 function evaluation in the ovary. The role of miRNA-21 in carcinogenesis has been studied, it has been found that miRNA-21 expression is raised in most of tumor. In the ovary tissues miR-21 is controlled after in vivo hCG/LH activation, intensely expressed in human granulosa cells affecting granulosa cells growth and maturation [32, 41, 64]. Granulosa cells apoptosis happens by the omission of miR-21 through particular inhibitors; therefore miR-21 acts for granulosa cells maintenance in the follicles prior to the ovulation [41]. Notably, in vivo miR-21 action blockade through using blocking oligonucleotide into the ovarian bursa stopped ovulation [9, 41]. Also In vivo inhibition of miR-224 can lead to impaired ovulation [55]. Similar studies demonstrated inactivation of specific associated miRNAs can imitate the Dicer-knockout phenotype in ovaries, implicating miRNAs critical functions in ovulation. Lately, miR-125b has been recognized as a downstream influencer of the androgen receptor in mouse granulosa cells [65]. Androgens upregulate miR-125b expression in granulosa cells, and blocks apoptotic pathways leading to increased granulosa cell maintenance [65, 66].

### Corpus luteum miRNA

The corpus luteum (CL) is made following ovulation and on account of the differentiation of mural granulosa cells. The corpus luteum is an endocrine gland works as hormonal provision for primary pregnancy and therefore is reduced in the lack of pregnancy, and also nearly prior to the beginning of the subsequent menstrual cycle. Lately, one research has pointed to the query of miRNA interference in luteal function, though many further research has recognized a number of significant miRNAs comprising miR-17-5p, let-7b, miR-125, miR-378, and miR-122 inside luteal tissues at disparate physiological phases (CL development, pregnancy, and regression). Through line of investigations miRNAs interfering regulatory mechanisms in the CL practice has been perceived. Since thorough lack of Dicer is lethal to embryos, therefore the scientists produced a Dicer hypomorphic mouse (dicerhypo) produces lower amounts of Dicer and lives up to adulthood. The lack of CL in Female dicer hypo mice leads to infertility, a disorder with frequent abortions in females [8].

Researchers suggest that the luteal tissue vascularization absence originates from the lack of some miRNAs (like miR-17-5p and let-7b) that affect the anti-angiogenic elements, tissue inhibitor of metalloproteinase-1 (timp1) miR-17-5p and let-7b replacement by straight transfection into the pregnant dicerhypo mice bursa may somewhat save the mice by enhancing vascularity and raising progesterone amounts. Though, one dose administration could not be able to keep pregnancy, the interference of other factors is possible [8]. Also, a group of researchers applied a bovine model to investigate miRNA expression alterations affecting CL function in periods of higher levels of progesterone compared to the time that CL bears regression, noticeable changes in miRNA production were perceived [67]. Since miRNA-378 cause the CL up-regulation with no recession further studies need to be conducted so as to recognize the likely character of this miRNA in CL up-keep. Based on preceding investigations, miRNA-378 acting in apoptosis through dropping the expression of interferon gamma 1 receptor (IFNGR1) gene. Consequently, Quantitative RT-PCR experimentations proved the link between higher levels of IFNGR1 mRNA and miR-378 with CL in the mid and late phases of CL upkeep, though minor levels of miR-378 in CL regression. Increased protein expression supported the possibility of post-transcriptional regulation of IFNGR1, however no change in IFNGR1 mRNA amounts within luteal regression has been seen [67]. CYP19, recognized as an estrogen-synthesizing aromatase enzyme, expressed nearly prior to ovulation and beginning of CL production. miR-378 can lead to CYP19 downregulation in porcine granulosa cells [68]. As well, a hairpin pre-miRNA inside the intron number 1 of the peroxisome proliferator-activated coactivator γ-1β (PGC-1β) gene produces miR-378 and miR-378 [69]. Although we found no reports of PGC-1β activity in ovarian cells, genetic alterations of the gene PGC-1α recognized in women with PCO [70, 71]. Therefore, in vivo studies are essential for confirming the biological activity of this miRNA because most cases of miR-378 in ovarian function have been reported by in vitro experiments.

### miRNA in the oocyte

With oocyte growth in the follicle, maternal mRNA and proteins are collected, relied on even fertilization and the embryonic genome activation. miRNA biogenesis pathway involved genes expression status in the mammalian oocytes and cleavage time embryos was examined through PREGER databank [72, 73]. Revealed by research, Drosha, DGCR8 and Dicer mRNA levels, as vital compartments of the miRNA pathway, upsurge throughout mammalian oocyte development. Most of oocytes and early embryos expressing miRNAs biogenesis pathways are unresponsive to α-amanitin, an RNA polymerase enzyme, leads to chiefly raised miRNAs expression in cleavage-time embryos [73]. Owing to the finding of distinctive alterations in miRNA biogenesis in the oocyte, our comprehension of oocyte biology has been impacted, herein we tried to discuss miRNA expression alterations and their significance in the human oocyte understanding. Typically, mouse oocytes, are studied for oocyte miRNA research, which above full-length Dicer expression, as well generates a particular oocyte figure that is not indicated in other species [74]. The mice and rats dicer gene consists of an MT-C retrotransposon promoter inside intron 6, which resulted in the yield of a short amino-terminal isoform of dicero (Dicero), specifically essential in the endogenous siRNA production for mouse oocyte function [74]. Therefore, it is predicted that the bulk of RNA species created in the mouse oocyte are consist of siRNAs and miRNAs. Dicer/AGO2 pathway produce double-stranded siRNAs, however dissimilar to miRNAs, siRNAs are not relied on DGCR8. Dicer Omission bases infertility in females and atypical metaphase spindles in mouse oocyte development, like parent AGO2-zp3-cKO and Dicer-zp3-cKO mice, therefore approving preceding research [7, 74, 75]. Conversely, DGCR8-zp3-cKO oocytes develop and fertilize with no obvious aberrations [76]. These preceding investigations confirmed the necessity of ago2 and dicer signaling communications for mouse oocyte development but not dgcr8. The origin of metaphase failure through maturing is unidentified, it might because of particular cytoskeletal regulation and cell cycle progression interfered genes directing endogenous siRNAs [42, 74, 77]. Majority of oocyte-related miRNA investigations have been conducted in rodent species expressing a shortened form of dicer with no expression in other species. Consequently, it is worth to inspect the siRNA and miRNAs corresponding functions in further species. Line of research led on bovine oocytes and as well other species, showing that miRNAs perform practically in oocytes developments [78, 79]. Similar investigation studied the function of miR-212 in the FIGLA regulation. FIGLA is an oocyte-related transcription factor that is necessary for follicle maturation and the synchronized expression of the zona pellucida proteins, ZP2, ZP3, and ZP1 [80]. According to number of research investigating the influence of miRNAs in the FIGLA expression regulation, studying the possible connection spots in the 3′UTR of bovine FIGLA mRNA by means of MicroInspecto software. This examination recognized miR-212 as a controller of FIGLA expression in bovine germinal cells. miR-212 and miR-132 expressions in tandem are co-regulated in granulosa cells after LH-rise in follicles [32].

Examining the expression outline of miR-212 of bovine tissues presented that it is pronouncedly expressed in the oocytes germinal vesicle with an inclination to escalation in the cleavage time of the embryo till the eight-cell stage, when the cow embryo experiences this transmission from the mother zygotic gene regulation. This expression outline is in consistence with other miRNAs expected to regulate transcript throughput in maternal-zygote transmission, consisting Zebrafish miR-430 [78], Xenopus miR-427[79], and mouse miR-290 [81]. The miR-212 expression in embryo and oocyte is counter-correlated with the expression of FIGLA, representing a possible miR-212 negative controlling function [63]. By means of FIGLA in miR-212 transfected cell culture models it has been displayed that miR-212 connects to the FIGLA mRNA 3′UTR head. miR-212 transfection simulates declining in FIGLA protein expression in the stage of eight-cells bovine embryos, representing the function of miR-212 in the control of FIGLA transcription factor [63]. Comparable research has merged miR-181a and miR-196a in the regulation of maternal oocyte related NPM2 and NOBOX genes [82, 83].

Up to the present time, some investigations have been accomplished concerning miRNA expression changes in human oocytes. In research, human MII oocytes genes expression was studied compared to human blastocysts [84]. By means of "Genome Survey Microarray", oocytes biosynthetic genes miRNA amounts and blastocysts were comparatively examined. Candidate genes narrow down was made, together with "housekeeping" genes as well human embryonic stem cells genes identified. A category of miRNAs has been derived from the nonhuman primate PREGER gene expression databank [73]. Similarly, non-human mammals, Drosha and Dicer gene yields were identified in human oocytes as well blastocysts. It has been found that, three nucleotides repeat gene 6B (TNRC6B), is a piece of the RISC complex, has been mainly expressed in oocytes. Exportin 5 (XPO5), responsible for transferring pre-miRNAs to the cytoplasm, was pronouncedly expressed in the blastocyst. Dicer, Drosha, Gem (nuclear organelle)-associated protein 5 (GEMIN5), and TNRC6B elevation in the oocyte have been comparable to what identified between mouse oocytes and blastocysts [72, 73, 85]. MOV10 mRNA, involved in human oocytes RISC complex [86] was untraceable in non-human primates however markedly increased in the human blastocyst [84]. PIWIL1 miRNA, part of the RISC assembly, affects germinal stem cell upkeep in Drosophila, nevertheless has not been discovered neither in human samples nor non-human primates [73, 84].

## miRNAs and urogenital disorders

Lately several groups of researchers have stated that disparate expression and disarray of miRNAs are attributed to ovarian diseases, like POF, PCOS and ovarian cancer [87, 88].

### miRNAs and POF

POF is ovary related disorder caused by various factors and is mostly reported as the incidence of amenorrhea, hypergonadotropism and hypoestrogenism in women below the age of 40 [89]. Investigations conducted on ovarian tissue and plasma recognized interfered miRNAs in the creation of POF condition. Dong et al. found that the miR-22-3p plasma level was declined in Han Chinese patients POF group in comparison to the control group. It was identified that miR-22-3p expression was associated with decreased ovarian maintenance [90]. Based on preceding research, different miRNAs in plasma of women with POF and women with healthy cycling are presented, with different roles in affecting signaling pathways [87]. In addition, Kuang et al. recognized 63 increased and 20 decreased miRNAs in ovarian biopsies of 4-vinylcyclohexene diepoxide (VCD)-prompted mice POF mockups in comparison to the biopsies of healthy mice [16]. Advanced studies proved that the miR-29a and miR-144 downregulation in POF samples and their potential function in prostaglandin synthesis regulation through directing PLA2G4A, however increased expression in variety of miRNAs, including, miR-151, miR-672, miR-190 and miR-27b, affects the hormonal stimulating and apoptosis [16]. Recently studies specify that single-nucleotide polymorphisms (SNPs) miRNA are connected with vulnerability degree of disease. In A study regarding miRNA polymorphism the connection between joint genotypes and haplotypes of miR-146aC > G, miR-196a2T > C, and miR-499A > G in Korean women with POF has been recognized; based on their findings the miR-146a and miR-196a2 transcriptional aberration persuaded by SNPs miRNA with potential function in POF development [91]. Effective miRs in immunopathology of POF are inclouding.

#### Mir23a

Since both TRERNA1 and miR-23a expression are varied in POF, probably TRERNA1 and miR-23a cooperatively act to control granulosa cell apoptosis [92]. Since, Granulosa cells are in charge of producing steroids and LH receptors to protect and ensure the ovaries healthy work, and profuse granulosa cell apoptosis pay to the creation of POF through decreased ovarian healthy function [93]. Overexpression of TRERNA1 can lead to decreased apoptosis rate in KGN cells. Thus, TRERNA1 probably pay to POF by stopping apoptosis in granulosa cells, and increasing its expression can be effective in the treatment of POF. Irregular and differential expression of miRNAs could be considered as possible biosignals for variety of diseases [94]. For instance, miR-21 overpowers the ovaries granulosa cells proliferation through directing SNHG7 in early ovarian insufficiency coupled with PCO (polycystic ovary syndrome) [94]. Also miR-23a can encourage apoptosis in granulosa cells, representing its contribution in POF [95]. miR-23a overexpression inhibits the SIRT1 expression, decline in the SIRT1 expression, hinders the p-ERK1/2 expression which leads to rise in apoptosis of GCs, TRERNA1 can suppress the miR-23a which is promoting KGN cell apoptosis [96, 97].

#### miR-146b-5p

Based on conducted research, miR-146b-5p indicate an encouraging influence on early ovarian failure in mice. miR-146b-5p cooperates with lncRNA DLEU1, a significant key in ovarian cancer development, consequently heightened DLEU1 expression and lowered miR-146b-5p expression in POF. DLEU1 cooperates with MiR-146b-5p which presented in both KGN cells nuclei as well cytoplasm samples. Though, DLEU1 encouraged cell apoptosis and abridged the miR-146b-5p preventive properties on cell apoptosis [98]. Granulosa cells (GCs) as follicular somatic cells are in charge for excreting steroid derivatives and supplementing vital nutrients for follicles generation [93, 99]. Accordingly, GC unhealthy function and risen apoptosis results in POF progression. Based on research, DLEU1 pronouncedly expressed in POF patients, caused GC apoptosis. Since, DLEU1 heightened expression in POF can support syndrome development through intensifying cell apoptosis, therefore DLEU1 suppression could probably benefit in POF treatment. MiR-146b-5p has been stated to contribute in mouse POF through overpowering γH2A phosphorylation and disabling Dab2ip/Ask1/ p38-Mapk pathway [100]. miR-146b-5p overexpression causes reduction in G cells apoptosis. Thus, miR-146b-5p performs a protecting role in POF through stopping cell apoptosis therefore might control the miR-146b-5p expression, and might be worked in the clinic for POF treatment. DLEU1 can cooperate with miR-146b-5p, also DLEU1 is detectable in GCs nucleus and cytoplasm samples and more remarkably, DLEU1 and miR-146b-5p are not affected by the expression of each other, however DLEU1 repressed the miR-146b-5p apoptotic effect. The lncRNAs are responsible for sponging miRNAs to stop their work nevertheless not affect expression status, DLEU1 with sponging miR-146b-5p could promote apoptosis in GCs, in so doing encouraging POF [98].

#### miR-144-5p

miR-144-5p expression allegedly has been utilized as a predictive bio-signal for many cancers including breast esophagus [101], gastric [102]. Contrary to mentioned findings, one research stated that decrease in expression of miR-144-3p and miR-144-5p recurrently detected in bladder cancer cells and silencing miR-144-5p stopped tumor cell growth through encouraging cell cycle arrest [103]. These miRNAs function intricately in a range of biological practices so as to uphold body [104]. Likewise, exosome miR-144-5p in BMSCs is capable to object PTEN, CTX-damaged GCs apoptosis involved protein. PTEN adversely influences the PI3K/AKT signaling inducing apoptosis or cell cycle arrest at the G1 phase [105] granulosa cells apoptosis ultimately resulted in premature ovarian failure (POF) then infertility.

#### miR-15b

miR-15a Overexpression results in growth prevention of and aging mouse ovary granulosa cells [106]. In a research on mice POI, it was discovered that miR-15b heightened expression causes POI and endogenous α-Klotho mRNA suppression together with instigating the function of lower hand transforming growth factor β1 (TGFβ1)/SMAD pathway [107]. As well, in cultivated mice granulosa cells treated with elevating dosages of cisplatin, it has been revealed that miR-125a-5p triggered granulosa cells apoptosis through dropping signal transducer and activator of transcription 3 (STAT3). Since STAT3 is worked in several reproductive purposes through transducing signs responding to growth factors and cytokines, this discovery delivers different visions toward POI comprehension [108].

#### miR-146a

**Fig. 1:**
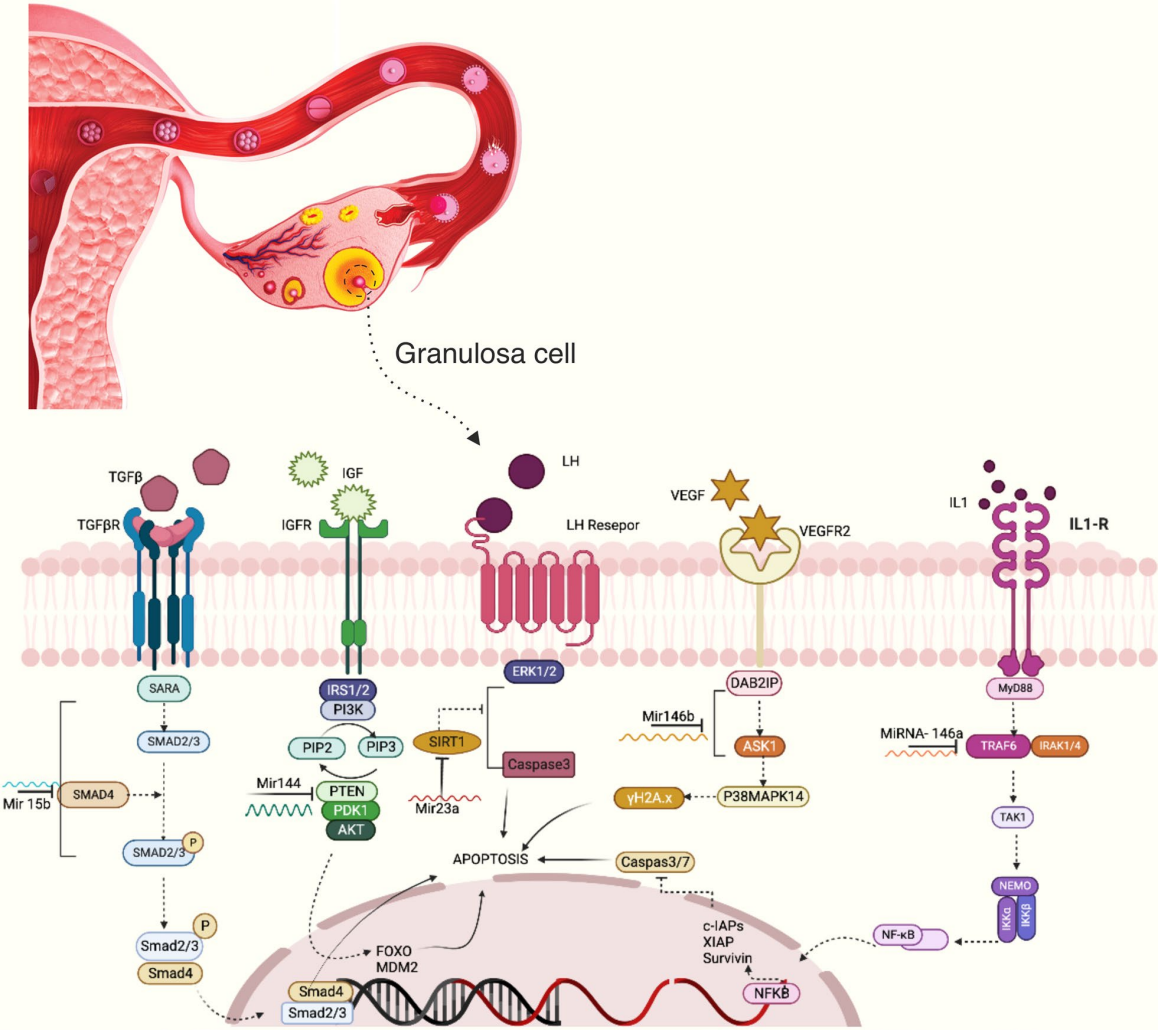
According to the figure, several miRs induce apoptosis in granulosa cells by targeting different molecules and signaling pathways. miR-146a activates apoptosis by targeting TRAF 6. Downstream signaling of TRAF6 leads to the production of anti-apoptotic molecules such as survivin, XIAP, and cIAPs which are inhibitors of caspase 3 and 7. In another way, miR-146b can induce apoptosis by inhibiting DIAB2 expression. Mir23a by targeting SIRT1 leads to the activation of caspase 3 and apoptosis. miR-144 by targeting PTEN leads to the activation of MDM2 and FOXO, and apoptosis occurs. miR-15 leads to apoptosis by inhibiting SMAD4

Interleukin (IL)-1 receptor-attributed kinase (IRAK1) and tumor necrosis receptor-associated factor 6 (TRAF6), are two important scaffold/ adaptor/proteins in the IL-1 and Toll-like receptor (TLR) signaling pathway, identified as positive regulators for nuclear factor (NF)-κB function, expressed by IκBα and IRAK1 and TRAF6 phosphorylation are assumed to be directed by miR-146a as a part of the NF-κB-prompted negative feedback [109, 110]. NF-κB has been confirmed to be engaged in the other biological processes [111], immune reactions, apoptosis and inflammation. The caspase signaling cascade is an imperative pathway for apoptosis, and caspase-8 and caspase-9 activation leads to cell apoptosis, with cleaving the cell apoptosis executor caspase-3 to degrades the substrate poly (ADP-ribose) polymerase (PARP) [112]. Consequently, it was presumed that miR-146a pays to the ovarian granulosa cells apoptosis through caspase cascade by directly targeting IRAK1 and TRAF6 [113] (Fig. 1 and Table1).

**Table 1:** Type of effective MiRNAs in POF

MiRNA	Function in POF	Refs
1. Mir23a	Both TRERNA1 and miR-23a were downregulated in POF	[92–97]
2. miR-146b-5p	Increased DLEU1 expression and decreased miR-146b-5p expression were observed in POF. DLEU1 promoted cell apoptosis and reduced the inhibitory effects of miR-146b-5p on cell apoptosis	[93, 98–100]
3. MiR-144-5p	exosomal miR-144-5p in BMSCs was able to directly target PTEN, a protein involved in the apoptosis of CTX-damaged GCs. PTEN has been shown to negatively regulate the PI3K/AKT pathway and induce apoptosis or cell cycle arrest at the G1 phase	[101–105]
4. miR-15b	overexpression of miR-15b induces POI by silencing the endogenous α-Klotho mRNA and stimulating the activity of the downstream transforming growth factor β1 (TGFβ1)/SMAD pathway	[106–108]
5. miR-146a	miR-146a contributes to the apoptosis of ovarian granulosa cells via the caspase cascade by directly targeting IRAK1 and TRAF6	[109–113]

## Conclusions

POF is a prevalent medical condition accompanying intricate molecular mechanisms. Herein we tried to review miRNAs as a group of critical regulatory elements after transcription in the development of POF. With regard to ability of an individual single miRNA in repressing expression of numerous genes, and probability of a single gene expression adjustment by several miRNAs, copious miRNAs are expressed in GC interfere in the maintenance and healthy practice of ovarian follicles, including ovulation, atresia, and ovarian steroidogenesis through directing certain molecules and manipulating variety of signaling pathways, like TGFB. Furthermore, miRNAs interfere decisively in female reproductive diseases, like POF, GCT, and PCOS, by influencing GC.

## Prospects and clinical applications of miRNAs in the treatment of premature ovarian failure

Due to the advances made in genomic and proteomic sciences, more comprehensive information about more miRNAs and their role in human health, diseases and also treatment has been provided. miRNAs have always had important effects in important cellular activities including transcription, gene translation and epigenetics. In this article, we have presented several reports on the effect of microRNAs on the important pathological processes of premature ovarian failure. Knowing the role of each of these miRNAs in the ovary can help in the early diagnosis of the disease and choosing the best treatment for the patient. As a result, conducting other specific researches on the types of effective miRNAs and their mechanism of action in the ovary, can help in timely diagnosis and treatment of the ovarian disease including premature ovarian failure.

## Data Availability

Not applicable.

## Abbreviations

ILs: Interleukins
NK cells: Natural killer cells
POF: Premature ovarian failure
SMAD: Small mothers against decapentaplegic
TGFbeta: Transforming growth factor-beta
PTEN: Phosphatase and tensin homolog
MiRNA: MicroRNA
PCOS: Polycystic ovary syndrome
TRAF6: Tumor necrosis factor receptor associated factor 6
IRAK1: Interleukin-1 receptor associated kinase 1
SiRNA: Small interfering RNA
ART: Assisted reproductive technologies
ALKs: Activin receptor-like kinases
LHCGR: Luteinizing hormone receptor
LH: Luteinizing hormone
CGC: Cumulus granulosa cells
